# Effect of probiotics and acidifiers on feed intake, egg mass, production performance, and egg yolk chemical composition in late-laying quails

**DOI:** 10.14202/vetworld.2024.462-469

**Published:** 2024-02-23

**Authors:** Widya Paramita Lokapirnasari, Mohammad Anam Al-Arif, Nanik Hidayatik, Aldhia Safiranisa, Dynda Febriana Arumdani, Amadea Inas Zahirah, Andreas Berny Yulianto, Mirni Lamid, Tabita Dameria Marbun, Ertika Fitri Lisnanti, Zein Ahmad Baihaqi, Aswin Rafif Khairullah, Shendy Canadya Kurniawan, Erlycasna Beru Sembiring Pelawi, Abdullah Hasib

**Affiliations:** 1 Division of Animal Husbandry, Faculty of Veterinary Medicine, Universitas Airlangga, Jl. Mulyorejo, Kampus C Mulyorejo, Surabaya 60115, East Java, Indonesia; 2 Division of Veterinary Basic Medicine, Faculty of Veterinary Medicine, Universitas Airlangga, Jl. Mulyorejo, Kampus C Mulyorejo, Surabaya 60115, East Java, Indonesia; 3 Master of Veterinary Agribusiness, Faculty of Veterinary Medicine, Universitas Airlangga, Surabaya 60115, East Java, Indonesia; 4 Faculty of Veterinary Medicine, Universitas Wijaya Kusuma Surabaya, Jl. Dukuh Kupang XXV No.54, Dukuh Kupang, Dukuh Pakis, Surabaya 60225, East Java, Indonesia; 5 Animal Nutrition Laboratory, Kyungpook National University, Sangju 37224, Korea; 6 Program of Animal Husbandry, Faculty of Agriculture, Universitas Islam Kadiri, Kediri. Jl. Sersan Suharmaji 38, Kediri 64128, East Java, Indonesia; 7 Research Center for Animal Husbandry, National Research and Innovation Agency (BRIN), Bogor Jl. Raya Jakarta Bogor 32 Cibinong 16915, West Java, Indonesia; 8 Master Program of Animal Sciences, Department of Animal Sciences, Specialisation in Molecule, Cell and Organ Functioning, Wageningen University and Research, Wageningen 6708 PB, Netherlands; 9 School of Agriculture and Food Sustainability, The University of Queensland, QLD, Queensland, Australia

**Keywords:** acidifiers, feed additive, health, performance, probiotic

## Abstract

**Background and Aim:**

Probiotics can be used as an alternative to antibiotic growth promoters because antibiotics are prohibited worldwide. This study investigated the potential combination of probiotics and acidifiers to improve feed intake, productive performance, egg mass, and egg yolk chemical composition of late-laying quail for the health of humans who consume quail products.

**Materials and Methods:**

One hundred laying quails were divided into 4 × 5 treatments, with each group consisting of five replications. The adaptation period was 2 weeks, and the treatment was continued for 4 weeks. Probiotics and acidifiers were added to drinking water and incorporated into the diet. Feed and water were provided *ad libitum*. Treatment duration (1 week, 2 weeks, 3 weeks, and 4 weeks) and additional feed treatment (control, probiotic 2% + 0.5% acidifier, probiotic 2% + 1% acidifier, probiotic 4% + 0.5% acidifier, and probiotic 4% + 1% acidifier, respectively).

**Results:**

Significant differences (p < 0.05) were observed in feed intake, quail day production, feed efficiency, egg mass in laying quails, and the chemical composition of egg yolk with probiotics and acidifiers in late-laying quails.

**Conclusion:**

The combination of probiotics and acidifiers can improve feed intake, production performance, egg mass, and egg yolk chemical composition in late-laying quails.

## Introduction

Currently, many regions of the world are testing alternative feed additives that can be used to alleviate the problems that stimulate antibiotic growth promoters (AGPs); therefore, research needs to be performed to find alternative ingredients to replace AGPs [1, 2]. Alternative ingredients to AGPs include acidifiers, probiotics, enzymes, herbal products for improving poultry health and production, microflora enhancers, and immunomodulators [3–6]. Yulianto and Lokapirnasari [7] successfully isolated lactic acid probiotics from the digestive tract of native chickens. Probiotics improve egg quality and production [8], livestock performance and chicken meat quality [9, 10], act as immunomodulators [11], and improve the quality of feedstuffs [12].

Probiotics, including *Pediococcus pentosaceus*, *Bifidobacterium* spp., *Lactobacillus casei*, and *Lactobacillus* *acidophilus*, are known to improve broiler performance [8, 9, 11, 13]. *P. pentosaceus* is a Gram-positive bacterium with a round shape that is non-motile, non-spore, and catalase-negative. In addition to lactic acids, it produces pediosin, a bacteriocin that can inhibit the growth of pathogenic bacteria such as *Listeria monocytogenes*, *Staphylococcus aureus*, *Escherichia coli*, *Vibrio alginolyticus*, *Pseudomonas stutzeri*, and *Aeromonas* in adequate quantities. The growth of *P. pentosaceus* requires nutrients, such as carbon, nitrogen, and minerals [14–16]. Acidifiers are one of the most common feed additives in industrial-scale poultry farms and are based on organic acids and salts [17]. Addition of an acidifier can improve digestibility and metabolism in livestock through increased digestive enzyme activity [18]. The use of organic acids can increase the number of lactic acid bacteria (LAB) in the ileum and cecum of broilers to maintain the balance of flora in the intestines [19]. Organic acids can improve poultry performance because they can change the pH of the digestive tract, thus changing the microbiome composition [20].

Therefore, the present study was conducted to investigate the effects of *P. pentosaceus* and *Bifidobacterium* spp. probiotics along with acidifiers on the feed intake, production performance, egg mass, and egg yolk chemical composition of late-laying quails.

## Materials and Methods

### Ethical approval

This study was approved by the Animal Care and Use Committee of Universitas Airlangga (No. 1.KEH.080.05.2023).

### Study period and location

This study was conducted from March 2023 to August 2023 at the Faculty of Veterinary Medicine, Universitas Airlangga.

### *In vivo* experiments

One hundred laying quails (*Coturnix coturnix japonica* strain) aged 28–34 weeks and weighing 185–200 g were divided into 4 × 5 treatments on a battery cage, each group consisting of five replications. The adaptation period was 2 weeks, and treatment was conducted for 4 weeks. Acidifiers and probiotics were applied through feed and drinking water, respectively. Feed and water were provided *ad libitum*. The acidifier used is a self-formulation consisting of organic acids, including fumaric acid and citric acid monohydrate.

The duration of treatment (a):

a^0^ = 1 week; a^1^ = 2 weeks; a^2^ = 3 weeks; and a^3^ = 4 weeks, respectively

Feed additive treatment (b):

b^0^ = control;

b^1^ = 2% probiotic (1% *P. pentosaceus* + 1% *Bifidobacterium* spp.) + 0.5% acidifier;

b^2^ = 2% probiotic (1% *P. pentosaceus* + 1% *Bifidobacterium* spp.) + 1% acidifier;

b^3^ = 4% probiotic (2% *P. pentosaceus* + 2% *Bifidobacterium* spp.) + 0.5% acidifier;

b^4^ = 4% probiotic (2% *P. pentosaceus* + 2% *Bifidobacterium* spp.) + 1% acidifier.

### Data collection

Data on feed intake were obtained from the amount of feed given minus the remaining feed. Feed intake (g/quail/day) = feed given (g/quail/day) – remaining feed (g/quail/day) was calculated every week during the 4 weeks of treatment using the following formula [21]:

Quail eggs are harvested every day. In addition, eggs are weighed to measure the egg mass. Egg production in quail day production (QDP) is calculated as the number of eggs produced per day divided by the total number of hens in the population and then multiplied by 100% using the following formula: QDP (%) = egg production a day/total of female quails 100.

Feed efficiency (%) was calculated by comparing the weight of the eggs produced to the amount of consumption, using the following formula: Feed efficiency (%) = Average egg mass (g)/feed intake (g) 100.

### Sample collection, sample preparation, and egg chemical composition analysis

Quail eggs were collected during the past 7 days of the study. The egg whites and yolks were separated and stored in a refrigerator every day. On the last day of egg collection, all 7-day egg yolks were mixed until homogeneous. The chemical composition was determined according to the Association of Official Analytical Chemists method [22].

### Statistical analysis

Quantitative data were analyzed using the analysis of variants method to determine differences between treatment groups. If there was a significant difference between treatment groups, Duncan’s multiple range test was performed at the 5% level to determine the best treatment results. Statistical Package for the Social Sciences 23.0 (IBM Corp., NY, USA) for Windows was used to perform the statistical analysis.

## Results

### Feed intake

A significant difference was observed in the average feed intake of laying quails treated with probiotics and acidifiers at different durations (p < 0.05). Table-1 lists the average feed intake (g/quail/day) with probiotics and acidifiers in laying quails. A high average feed intake was obtained during the 1^st^ week (18.00 g/head/day), which did not differ from the 4^th^-week treatment of probiotics and acidifiers (16.87 g/head/day), and the lowest feed consumption was obtained during the 3^rd^ week (15.91 g/head/day) and 2 weeks (16.58 g/head/day), which did not differ from the 4^th^-week treatment. A significant difference (p < 0.05) in feed intake was observed with the combined treatment of probiotics and acidifiers.

**Table-1:** Feed Intake average (g/quail/day) with probiotics and acidifiers in late laying quail.

Treatments	Duration				Average	p-value

	1 week (a0)	2 weeks (a1)	3 weeks (a2)	4 weeks (a3)		
b0	19.22^bc^ ± 0.32	14.38^a^ ± 5.14	14.94^ab^ ± 0.59	16.41^abcde^ ± 1.26	16.24^a^ ± 0.53	0.003
b1	16.44^abcde^ ± 1.10	18.48^bcde^ ± 2.55	16.11^abcde^ ± 2.93	16.02^abcde^ ± 0.09	16.76^ab^ ± 0.53	0.002
b2	18.89^cde^ ± 1.44	15.41^abc^ ± 1.96	15.84^abcd^ ± 2.28	15.89^abcd^ ± 0.93	16.51^a^ ± 0.53	0.003
b3	17.19^abcde^ ± 0.84	18.63 ^cde^ ± 3.55	17.44^abcde^ ± 2.09	19.55^e^ ± 1.08	18.20^ab^ ± 0.53	0.002
b4	18.28^bcde^ ± 1.93	15.99^abcde^ ± 2.58	15.24^abc^ ± 0.97	16.49^abcde^ ± 1.80	16.50^a^ ± 0.53	0.002
Average	18.00^b^ ± 0.47	16.58^a^ ± 0.47	15.91^a^ ± 0.47	16.87^ab^ ± 0.47		

Different superscripts in rows and columns indicate significant differences (p < 0.05)

### QDP

The average QDP (%) with additional probiotics and acidifiers in late-laying quail is shown in Table-2. The addition of probiotics and acidifiers to late-laying quails with different durations showed a significant difference (p < 0.05) between QDP treatment group. High QDP results were found at 3 weeks (63.73) and 4 weeks (64.31), which did not different from the 2 weeks (62.29). The lowest QDP value was 60.85 at 1 week. There was a significant difference (p < 0.05) in QDP in the combination of probiotics and acidifiers. The highest QDP values were found in 2% probiotic treatment and 0.5% acidifier (b1) and 4% probiotic and 0.5% acidifier b3, reaching 70.48% and 68.79%, respectively. The lowest QDP value (52.53%) was observed in the control group without the additional probiotics.

**Table-2:** Average quail day production (%) with probiotics and acidifiers additional in late laying quail.

Groups	Duration				Average	p-value

	1 week (a0)	2 weeks (a1)	3 weeks (a2)	4 weeks (a3)		
b0	54.61^bc^ ± 0.63	53.45^abc^ ± 0.79	52.68 ^ab^ ± 0.59	49.41^a^ ± 2.27	52.53^a^ ± 0.79	0.001
b1	69.96^fg^ ± 0.08	70.00^fg^ ± 1.65	70.54 ^fg^ ± 1.78	71.43^g^ ± 0.00	70.48^d^ ± 0.79	0.005
b2	57.50^bc^ ± 2.70	63.57^e^ ± 3.03	66.43^efg^ ± 1.75	66.97^efg^ ± 6.76	63.61^c^ ± 0.79	0.005
b3	66.11^ef^ ± 7.18	67.32^efg^ ± 5.29	70.72^fg^ ± 1.43	71.01^fg^ ± 3.78	68.79^d^ ± 0.79	0.010
b4	56.07^bc^ ± 2.13	57.14^bc^ ± 4.03	58.33^cd^ ± 2.38	62.74^de^ ± 1.12	58.57^b^ ± 0.79	0.001
Average	60.85^a^ ± 1.58	62.29^ab^ ± 1.58	63.73^b^ ± 1.58	64.31^b^ ± 1.58		

Different superscripts in rows and columns indicate significant differences (p < 0.05)

### Feed efficiency

Table-3 lists the average feed efficiency (%) for late-laying quails supplemented with probiotics and acidifiers. A significant difference was observed in treatment duration (p < 0.05). The highest feed efficiency values were observed after 4 weeks with 2% probiotic and 0.5% acidifier (a3b1). b1 treatment for 1, 2, 3, and 4 weeks did not show significantly different results. The b2 treatment for 2, 3, and 4 weeks, the b3 treatment for 3 and 4 weeks, and the b4 treatment for 3 and 4 weeks yielded good results. The lowest feed efficiency values (b0) were observed in the control treatment.

**Table-3:** Feed efficiency average (%) with probiotics and acidifiers additional on late laying quail.

Groups	Duration				Average	p-value

	1 week (a0)	2 weeks (a1)	3 weeks (a2)	4 weeks (a3)		
b0	30.62^a^ ± 1.19	41.76^bcd^ ± 14.33	37.23^abc^ ± 2.78	29.41^a^ ± 1.50	34.75^a^ ± 1.65	0.001
b1	48.98^de^ ± 3.87	46.70^cde^ ± 8.25	53.32^e^ ± 13.33	53.43^e^ ± 2.29	50.60 ^c^ ± 1.65	0.001
b2	35.03^ab^ ± 2.65	48.69^de^ ± 5.97	51.89^de^ ± 6.58	51.62^de^ ± 4.88	46.80^bc^ ± 1.65	0.005
b3	44.04^bcde^ ± 2.85	41.37^bcd^ ± 10.68	46.89^cde^ ± 6.79	45.20^bcde^ ± 7.53	44.36^b^ ± 1.65	0.001
b4	36.00^abc^ ± 4.29	41.96^bcd^ ± 3.51	44.56^bcde^ ± 2.26	45.88^bcde^ ± 4.15	42.09^b^ ± 1.65	0.001
Average	38.93^a^ ± 1.47	44.09^b^ ± 1.47	46.77^b^ ± 1.47	45.10^b^ ± 1.47		

Different superscripts in rows and columns indicate significant differences (p < 0.05)

### Egg yolk chemical composition

The combination of probiotics and acidifier was resulted in better nutrient values than the control. A significant difference (p < 0.05) was observed in the dry matter composition of egg yolks between the treatments. There was no significant difference in the ash composition of egg yolk (p > 0.05) between the treatments. There was a difference in crude protein composition of egg yolk (p < 0.05) between treatments. The highest crude protein content in egg yolk was observed in the b2 and b1 treatments. The composition of the ether extract in egg yolk differed between the treatments (p < 0.05). The lowest extract ether composition was observed in the b3 and b4 treatments, followed by the b2 and b1 treatments. The use of probiotics and acidifiers in late-laying quail showed a significant difference (p < 0.05) in the crude egg yolk fiber composition. The lowest crude fiber content was found in the b1, b2, b3, and b4 treatments, whereas the highest crude fiber content was found in the b0 treatment. A significant difference (p < 0.05) was observed in the egg yolk NFE composition between the treatments. The highest NFE composition was observed in b4, b3, b2, and b1. There was no significant difference in the metabolic energy composition of egg yolk (p > 0.05) between the treatments. A significant difference (p < 0.05) in egg yolk carbohydrate composition was observed in the combination of probiotics and acidifier usage in late-laying quail. High and low carbohydrate compositions were found in b3 and b4, followed by b1 and b2 and b0 treatments. The use of probiotics and acidifiers in late-laying quail showed a significant difference (p < 0.05) in egg yolk organic matter (OM) composition. High OM composition was found in the b1, b2, and b3 treatments.

## Discussion

### Feed intake

Due to the ban on antimicrobial growth promoters in a number of production systems, organic acids and probiotics have been widely used in the poultry sector to improve the performance and health of hens. Fumaric, formic, lactic, butyrate, propionic, and citric acids and their salts have been extensively investigated and used [23]. Table-1 lists the average feed intake (g/quail/day) with probiotics and acidifiers in laying quails. Acidifiers have the potential to improve nutrition utilization, alter gut pH, and reduce the growth of dangerous microorganisms in the digestive system. This result agrees with a previous study by Cao *et al*. [24] in which acidifier addition improved the daily feed intake and had significant interaction effects with probiotics on feed intake and egg weight. This result also agrees with Haque *et al*. [25] and Fascina *et al*. [26], who reported that broilers fed 0.5% citric acid or an organic acid mixture exhibited higher feed intake. The use of organic acids significantly enhanced egg production in layer hens [27, 28].

Karwanti *et al*. [29] showed that the use of 1% *P. pentosaceus* increased feed consumption by 7.7% compared to the control. The inhibition exclusion mechanism could explain the increase in serum total protein and albumin, where LAB can improve the utilization of dietary proteins by inhibiting pathogen growth, which reduces protein degradation into nitrogen and improves dietary protein efficiency and nutrient absorption [30].

### QDP

The addition of an acidifier to feed is useful for increasing the growth of probiotics in the digestive tract. The addition of organic acids can reduce the pH value in some parts of the digestive tract; this condition is favorable for the growth of beneficial bacteria and inhibits the growth of pathogenic bacteria that grow at high pH [31]. Table-2 shows the average QDP (%) with additional probiotics and acidifiers in late-laying quails. We found an interaction between the duration and the combination of probiotics and acidifier doses (p < 0.05) on the average QDP in laying quails treated with probiotics and acidifiers. The best interaction was found with 2% probiotic and 0.5% acidifier for 4 weeks (a3b1), which was not different with 4% probiotic and 0.5% acidifier for 4 weeks (a3b3), 4% probiotic and 0.5% acidifier for 3 weeks (a2b3), 2% probiotic and 0.5% acidifier for 3 weeks (a2b1), 2% probiotic and 0.5% acidifier for 2 weeks (a1b1) and 2% probiotic and 0.5% acidifier for 1 week (a0b1). The lowest QDP values (a3b0, a2b0, and a1b0) were observed in the control treatment.

Probiotics promote the growth of beneficial microorganisms while reducing the number of harmful bacteria in the intestinal microbial balance. Probiotic consumption can reduce the incidence of gastrointestinal disease by promoting good microorganisms [32]. Bifidobacteria are non-spore-forming anaerobic bacteria that produce antimicrobial protein substances, such as bacteriocins (bifidine and bifidosin B), lactic acid, and acetic acid, including *Bifidobacterium bifidum*. These substances have a positive effect in inhibiting the growth of some Gram-positive and Gram-negative bacteria *in vitro* so that later the development of pathogenic bacteria in the intestines can be suppressed and the poultry’s digestive system can run well [33–35].

### Feed efficiency

Probiotics are optimal if the pH of the chicken digestive tract is suitable for the growth of LAB. Optimal LAB growth suppresses the growth of pathogenic bacteria and decreases the number of intestinal pathogenic bacteria. Some pathogenic bacteria lead to optimal development of intestinal villi and improve nutrient absorption. LAB can produce antibacterial agents, including bacteriocins that suppress the growth of pathogenic microbes. Table-3 lists the average feed efficiency (%) with additional probiotics and acidifiers on late-laying quails. These results are in accordance with [12] research that probiotics (*L. acidophilus*, *L. casei*, *Lactococcus lactis*, *and Bifidobacterium* spp.) give positive results on production performance (body weight gain, body weight, feed consumption, feed efficiency, and feed conversion ratio), carcass production (carcass weight and percentage), and mortality in Peking ducks.

This study is in accordance with Agustono *et al*. [36], in which probiotics (*L. acidophilus*, *Lactobacillus plantarum*, and *Bifidobacterium* spp.) with 1.2 × 10^9^ concentration colony-forming units/mL showed positive results on growth performance (body weight, feed consumption, feed conversion ratio, carcass weight, and breast meat weight). Probiotics can increase broiler body weight and improve feed conversion ratio associated with increased feed efficiency [37], livestock growth performance, and feed efficiency [12, 21].

Karwanti *et al*. [29] reported an improvement in feed efficiency (62.92%) compared with the control (46.57%) using 1% *P. pentosaceus*. The addition of organic acids to broilers increased the body weight and feed conversion ratio compared to that without organic acids [38]. The highly acidic environment of the stomach contributes to the secretion of more gastric juice and pancreatic enzymes [39]. These main factors help digest food and absorb nutrients effectively [17].

### Egg mass

Table-4 lists the probiotic and acidifier treatments in late-laying quails at different durations. The use of probiotics in quail through feed and water is expected to improve the efficiency of feed and increase egg production [40]. The results of this study are also in line with research by Mas’ad *et al*. [41], who reported that the treatment group given additional probiotics in drinking water gave better results compared to the control group. The results of this study are in accordance with research conducted by Pradikta *et al*. [42], who reported that the value of egg mass increases as the amount of probiotics for laying hens increases. Probiotics that work in the small and large intestines can suppress pathogenic bacteria and stimulate the growth and activity of beneficial bacteria in the intestine, which can increase nutrient absorption [41].

**Table-4:** Egg mass average with probiotic and acidifiers addition on late laying quail.

Groups	Duration				Average	p-value

	1 week (a0)	2 weeks (a1)	3 weeks (a2)	4 weeks (a3)		
b0	5.88^bc^ ± 0.15	5.47^ab^ ± 0.08	5.55^ab^ ± 0.22	4.81^a^ ± 0.14	5.43^a^ ± 0.12	0.001
b1	8.02^fgh^ ± 0.15	8.48^h^ ± 0.43	8.30^gh^ ± 0.64	8.56^h^ ± 0.35	8.34^e^ ± 0.12	0.020
b2	6.59^cd^ ± 0.22	7.41^ef^ ± 0.29	8.11^fgh^ ± 0.18	8.20^fgh^ ± 0.91	7.58^c^ ± 0.12	0.010
b3	7.58^efg^ ± 0.82	7.44^ef^ ± 0.67	8.07^fgh^ ± 0.42	8.80^h^ ± 1.25	7.97^d^ ± 0.12	0.010
b4	6.52^cd^ ± 0.20	6.64^cd^ ± 0.53	6.78^de^ ± 0.31	7.51^efg^ ± 0.30	6.86^b^ ± 0.12	0.010
Average	6.92^a^ ± 0.11	7.09^ab^ ± 0.11	7.36^bc^ ± 0.11	7.57^c^ ± 0.11		

Different superscripts in rows and columns indicate significant differences (p < 0.05)

Dietary probiotic supplementation enhanced the daily feed intake, egg production rate, and body weight of ducks, whereas diet acidifier increased the daily feed intake compared with the control. Dietary intake of probiotics increases egg quality [24]. Organic acids produced during animal metabolism reduce the pH of the feed and digestive tract. Organic acids provide defense against pH-sensitive pathogens and improve nutrient digestibility and performance in poultry [43]. In the current study, the increase of egg mass features by probiotic addition in late-laying quail feed might be attributed to the effect on intestinal health, which resulted in improved feed efficiency. The result of the combination of probiotics and acidifiers in this study is in line with Ahiwe *et al*. [44], who reported that dietary supplementation with probiotics in animal diets increased the activities of A-amylase, trypsin, and chymotrypsin in duodenal chyme, which may result in higher protein digestibility and nutrient utilization.

### Egg yolk chemical composition

Quail eggs have substantially similar nutritional benefits to chicken eggs and are rich in antioxidants, minerals, and vitamins, providing more nutrients than other foods [45]. In this study, the results of nutrient quality analysis on quail eggs were obtained from a 7-day collection of quail eggs in the 4^th^ week. The chemical composition of quail egg yolk is presented in Table-5. Japanese quail eggs have a higher crude protein, crude fat, and mineral ash content per unit egg weight than chicken eggs and other popular poultry, such as pheasant and guinea fowl [46]. Another study by Oviawe *et al*. [47] reported that the proximate composition of whole quail eggs, namely, ash, carbohydrate, fat, protein, and moisture, were 1.06, 4.01, 9.89, 12.7, and 72.25 g 100 g^1^, respectively, and the energy obtained from whole eggs was 156.50 kcal 100 g^1^. The moisture, ash, and crude protein content of quail eggs in this study were higher than those reported by Tunsaringkarn *et al*. [48]. The chemical compositions [dry matter (%), crude protein (%), and ether extract content (%)] of the egg yolk of Japanese quails fed on experimental basal diets supplemented with lacto-sacc were 52.88–52.68, 29.67–29.25, and 63.45–63.34, respectively, and the supplemented with Thyme flowers were 52.78–52.94, 29.28–29.37, and 63.12–63.31, respectively.. Lacto-Sacc was obtained from Nicholasville Kentuky Biotechnology Center, Altech, USA. It mainly consisted of dried *Streptococcus faecium* fermentation product, dried *L. acidophilus* fermentation product, yeast culture (live *Saccharomyces cerevisiae* grown on media of ground yellow corn, diastatic malt, and cane molasses), dried *Aspergillus oryzae* fermentation extract, dried *Aspergillus niger* fermentation extract, and beta-glucan [49].

**Table-5:** Egg yolk average chemical composition with probiotic and acidifiers on late laying quails.

Chemical composition	b0	b1	b2	b3	b4	p-value
Dry matter (%)	83.28^a^ ± 0.22	84.39^b^ ± 0.01	84.15^b^ ± 0.30	84.01^b^ ± 0.76	83.42^a^ ± 0.15	0.001
Ash	3.26^a^ ± 0.17	3.22^a^ ± 0.57	3.31^a^ ± 0.24	2.95^a^ ± 0.05	3.21^a^ ± 0.06	0.480
Crude protein (%)	24.82^a^ ± 0.36	25.94^b^ ± 0.93	29.69^c^ ± 0.19	24.82^a^ ± 0.19	24.95^a^ ± 0.06	0.001
Extract ether (%)	15.06^c^ ± 0.49	11.61^b^ ± 0.19	11.24^b^ ± 1.19	9.81^a^ ± 0.86	10.13^a^ ± 0.47	0.001
Crude fiber (%)	30.47^b^ ± 5.12	26.30^a^ ± 0.38	26.31^a^ ± 0.46	26.21^a^ ± 0.86	25.82^a^ ± 0.40	0.039
Nitrogen free extract (%)	26.37^a^ ± 4.09	32.92^c^ ± 0.16	29.45^b^ ± 0.67	34.76^cd^ ± 0.05	36.13^d^ ± 0.38	0.001
Metabolizable energy (kcal/kg)	2954.87^a^ ± 185.44	2974.11^a^ ± 38.29	2945.06^a^ ± 66.32	2978.61^a^ ± 65.12	2933.50^a^ ± 39.43	0.956
Carbohydrate (%)	56.84^a^ ± 1.03	59.23^b^ ± 0.55	55.76^a^ ± 1.14	60.97^c^ ± 0.91	61.95^c^ ± 0.78	0.001
Organic matter (%)	80.02^a^ ± 0.04	81.17^b^ ± 0.56	80.84^b^ ± 0.04	81.06^b^ ± 0.71	80.20^a^ ± 0.21	0.004

Different superscripts in rows indicate significant differences (p < 0.05)

## Conclusion

A combination of probiotics (*Pediococcus* and *Bifidobacterium*) and an acidifier in late-laying quail positively affects feed intake, QDP, feed efficiency, egg mass, and the chemical composition of quail egg yolk. A combination of probiotics and acidifiers for 2–4 weeks can improve the performance of late-laying quail and egg mass and increase the nutrient value of eggs. Organic acids and probiotics have been identified as potential alternatives to dietary antibiotics to improve productive performance and increase egg quality in late-laying quails.

## Authors’ Contributions

WPL and ABY: Conception and design of the study. MAA, EBSP, and EFL: Analysis and interpretation of data. WPL, ABY, ARK, SCK, and NH: Investigation and data curation and reviewed the manuscript critically for important intellectual content. AS, DFA, and ML: Collected the sample and drafted the manuscript. EBSP, ABY, AIZ, TDM, ZAB, and AH: Analysis and data curation and critically revised the manuscript. All authors have read, reviewed, and approved the final version of the manuscript.
